# Metapopulation viability of an endangered shorebird depends on dispersal and human-created habitats: piping plovers (*Charadrius melodus*) and prairie rivers

**DOI:** 10.1186/s40462-016-0072-y

**Published:** 2016-03-15

**Authors:** Daniel H. Catlin, Sara L. Zeigler, Mary Bomberger Brown, Lauren R. Dinan, James D. Fraser, Kelsi L. Hunt, Joel G. Jorgensen

**Affiliations:** Department of Fish and Wildlife Conservation, Virginia Tech, Blacksburg, VA 24061 USA; U.S. Geological Survey, Woods Hole Coastal and Marine Science Center, Woods Hole, MA 02543 USA; School of Natural Resources, University of Nebraska, Lincoln, NE 68583 USA; Nongame Bird Program, Nebraska Game and Parks Commission, Lincoln, NE 68503 USA

**Keywords:** Conservation reliance, Dispersal, Disturbance, Extinction, Population viability analysis, Recolonization, Rescue effect, Successional processes

## Abstract

**Background:**

Many species are distributed as metapopulations in dynamic landscapes, where habitats change through space and time. Individuals locate habitat through dispersal, and the relationship between a species and landscape characteristics can have profound effects on population persistence. Despite the importance of connectivity in dynamic environments, few empirical studies have examined temporal variability in dispersal or its effect on metapopulation dynamics. In response to this knowledge gap, we studied the dispersal, demography, and viability of a metapopulation of an endangered, disturbance-dependent shorebird. We examined three subpopulations of piping plovers (*Charadrius melodus*) on the lower Platte and Missouri rivers from 2008–2013. High flow events from an upstream dam on the Missouri River in 2010 and 2011 allowed us to assess the effect of total habitat loss and the subsequent creation of new habitat associated with a large disturbance at one ‘natural’ study location. The other two sites within the metapopulation, which were maintained by anthropogenic activities (e.g., mining, development, habitat restoration), were largely unaffected by this disturbance, resulting in a controlled natural experiment.

**Results:**

High flow events were associated with increased emigration, decreased immigration, and decreased survival in the subpopulation that experienced high flows. Following the high flow event, immigration into that subpopulation increased. Dispersal rates among subpopulations were negatively correlated with distance. The metapopulation had a low probability of extinction over 100 years (0 %) under the current disturbance interval and associated dispersal and survival rates. However, persistence depended on relatively stable, human-created habitats, not the dynamic, natural habitat (47.7 % extinction probability for this subpopulation).

**Conclusions:**

We found that functional connectivity, as measured by the rate of dispersal among subpopulations, increased as a result of the high flow event in our study metapopulation. Plovers also increased reproductive output following this event. Although the study metapopulation had a low overall probability of extinction, metapopulation persistence depended on anthropogenically created habitats that provided a small but stable source of nesting habitat and dispersers through time. However, all subpopulations remained small, even if persistent, making them individually vulnerable to extinction through stochastic events. Given the highly dynamic nature of habitat availability in this system, maintaining several subpopulations within the metapopulation and stable sources of habitat will be critical, and this species will likely remain conservation-reliant.

**Electronic supplementary material:**

The online version of this article (doi:10.1186/s40462-016-0072-y) contains supplementary material, which is available to authorized users.

## Background

Habitat patchiness, spatial subdivision, and local extinction/recolonization dynamics are common in ecological systems, and many species are organized into metapopulations (i.e., a group of local subpopulations that inhabit discrete habitat patches but interact through dispersal) as a consequence [1]. Metapopulation theory and empirical studies of species distributed as metapopulations are valuable tools that inform conservation strategies for imperiled species, with applications in reserve design, corridor creation, and extinction risk estimation [2, 3]. As such, there has been a proliferation of theoretical and empirical studies of metapopulations and their properties.

Although basic metapopulation theory deals primarily with static systems, there is growing interest in its application to dynamic environments that more closely mimic natural systems [4]. Populations in dynamic systems experience local extinctions and colonization opportunities through both stochastic and deterministic (e.g., succession) processes [5, 6]. Metapopulation persistence in dynamic landscapes depends on the species’ demographic characteristics as well as underlying spatial (e.g., area, connectivity, patch size, patch quality) and temporal (e.g., turnover rate, extent, intensity) habitat properties [3, 7]. This interplay between species characteristics and the dynamic properties of a metapopulation and its habitat have important consequences for how disturbances and changing landscapes affect a species. Relatively few studies, however, have coupled empirical species data with dynamic landscape modelling to produce actionable management plans [3, 7].

The application of dynamic metapopulation models has clear connections with the management of imperiled species distributed as metapopulations naturally or due to anthropogenic fragmentation [8, 9]. As the study of metapopulations in dynamic landscapes matures, it is important to merge information on imperiled species ecology and landscape dynamism in empirical studies [7]. Such studies should relate manageable features of dynamic systems to species persistence, thereby providing qualitative and quantitative recommendations for the management of dynamic systems [3]. In addition, studies need to focus on less well-studied factors in dynamic systems (e.g., connectivity, variation in patch quality) [10], such that generalizations across variable landscapes can be made.

Structural and functional connectivity are fundamental characteristics affecting metapopulation persistence in dynamic landscapes because, as the spatial and temporal orientation of habitat changes, animals must locate habitat through dispersal [6, 10]. For instance, in dynamic, disturbed ecosystems, connectivity among subpopulations can allow (*i*) individuals to disperse away from areas experiencing a disturbance to refugia and (*ii*) surviving individuals to later recolonize recently disturbed or created habitat [6, 11, 12]. However, temporal changes in connectivity are rarely studied or modeled [10]. To fully understand metapopulation persistence in dynamic, disturbance-dependent systems, considering changes in connectivity due to natural or anthropogenic forces and developing metapopulation models that account for both spatial and temporal changes in connectivity is necessary [10].

We studied a disturbance-dependent animal that displays high site fidelity despite a capacity for long-distance dispersal, the piping plover (*Charadrius melodus*; hereafter ‘plover’), within a metapopulation on the Missouri and Platte rivers. Plovers in this area nest on a variety of habitats with a range of turnover and disturbance rates. We conducted this study (*i*) to determine the effect of disturbance and habitat turnover on dispersal and functional connectivity in the metapopulation; (*ii*) to use empirical data to compare the effects of human alterations to habitat turnover (i.e., subpopulation-specific habitat management) among dynamic and static metapopulation patches; and (*iii*) to make suggestions about the management of this system relative to functional connectivity and turnover to promote persistence.

## Methods

### Focal species

The piping plover is a migratory, precocial shorebird that nests on sparsely vegetated beaches on the Atlantic coast, Great Lakes, and Great Plains in the United States and Canada [13]. Plover adult annual survival averages 76 %, resulting in an approximately 5 years average lifespan [14]. Some birds breed in their first year post-hatch but do so approximately one month later than experienced adults [14]. Across their range, disturbances (e.g., high-water events on rivers, reservoirs, and alkali lakes; coastal storms) are critical for maintaining early successional habitat. Plovers were listed under the U.S. Endangered Species Act in 1986, primarily as a result of habitat loss and low reproductive output [15].

Plover populations are distributed as metapopulations throughout their geographic range, both naturally and as a result of anthropogenic landcover change. In the southern Great Plains, plover subpopulations on the Missouri River nest on sandbars [16] and sand and gravel mines and lakeshore housing developments in the lower Platte River floodplain [17]. Nesting areas are separated from other nesting areas by inhospitable expanses of channelized river, impoundments [15], development [17], and agricultural lands. In North Dakota, plovers disperse among subpopulations [18] that inhabit naturally isolated alkali lakes, riverine sandbars, and reservoirs [13], where flooding can have profound impacts on habitat and demography [19]. A similar pattern has been observed in Saskatchewan, where short- and long-term flooding has affected plover distribution and dispersal [20]. Outside of the Great Plains, plovers use ephemeral barrier island habitat created through storms and degraded through succession, erosion, and redevelopment [21, 22]. Thus, throughout their range, plovers are subject to local extinctions due to natural (e.g., flooding, succession) and anthropogenic (e.g., development) processes, relying on disturbance to refresh habitat and dispersal to recolonize newly available habitat.

Despite the metapopulation structure commonly observed for this species, relatively little is known about plover dispersal among subpopulations and across spatiotemporal scales or the implications of dispersal on metapopulation persistence (review in [14]), which could limit conservation options (e.g., [2]) for this Federally threatened species. One study related increased dispersal among nearby subpopulations to reproductive failure and flooding but did not evaluate the effect of disturbance and dispersal on persistence nor the effect of distance on connectivity [20]. An understanding of dispersal rates and connectivity based on distance could allow for improved regional models or forecasts of metapopulation persistence. Plover management has generally been specific to a subpopulation or a managing entity, increasing the difficulty of obtaining the appropriate information needed to understand metapopulation dynamics and managing the species at a metapopulation scale [15, 18].

### Study system

**Fig. 1:**
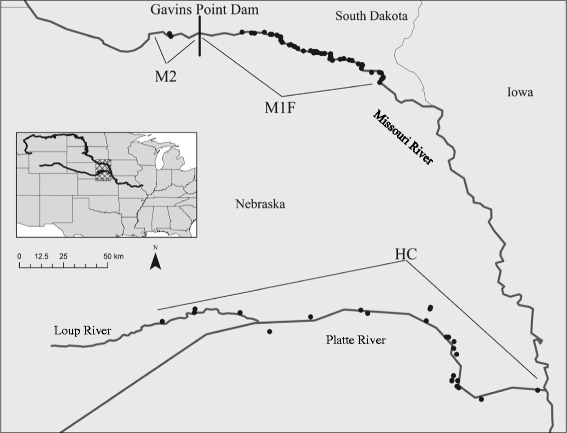
Map of the subpopulations on the lower Platte and Missouri rivers. Areas included those adjacent to sand and gravel mines (HC); Lewis and Clark Lake, a reservoir upstream of the Gavins Point Dam (M2); and the Gavins Point Reach of the Missouri River, downstream of the Gavins Point Dam (M1F). Nesting sandbars, sand and gravel mines, and housing communities within each subpopulation are represented with black circles

We studied three plover subpopulations along the lower Platte and Missouri rivers in Nebraska and South Dakota, U.S.A., from 2008 to 2013 (Fig. 1). Habitat used by the subpopulation at the confluence of the Platte and Missouri rivers is approximately 182 km and 241 km from habitat used by subpopulations at Gavins Point Reach and Lewis and Clark Lake, respectively. Nesting areas on the upstream section of the Gavins Point Reach are approximately 30 km from those in Lewis and Clark Lake. We chose these subpopulations and boundaries because they internally experienced similar conditions and were considered separate management units. In addition, plover movements were more frequent within subpopulations than among them (DHC and MBB, unpublished data).

Observations also support a metapopulation structure for these subpopulations. Dispersal events are commonly observed among our study subpopulations (result herein). However, during our study, no birds banded in our subpopulations were found breeding in populations north of our study area, and only one individual banded at Lake Sakakawea, North Dakota was known to breed in our study area (>780 km; DHC and MBB, unpublished data). We have also received limited reports of banded birds from our subpopulations nesting elsewhere. Most of these birds (8 total) bred in areas immediately adjacent (<5 km) to Lewis and Clark Lake in relatively small blocks of ephemeral habitat (DHC, personal observation). Two birds banded on Lewis and Clark Lake and the Gavins Point Reach were found nesting within another small population with more stable habitat in the upper Niobrara River near Spencer, Nebraska (>50 km). There was also limited movement between our subpopulations and another subpopulation on the central Platte River, near Kearney, Nebraska. Three birds banded on the central Platte were found nesting on the Gavins Reach and Lewis and Clark Lake (>260 km), and five were found nesting in the lower Platte subpopulation (>150 km). In contrast, no birds banded on Lewis and Clark Lake or the Gavins Point Reach were found nesting on the central Platte, and only one bird from the Lower Platte subpopulation was found nesting there (DHC and MBB, unpublished data). Most of these sightings were at locations adjacent to our metapopulation, and movements appeared to increase following high flow events. Therefore, the metapopulation we considered here was open, but the interchange of individuals between our subpopulations and others appeared to follow the same patterns as that within our metapopulation and likely did not have a strong effect on the observed metapopulation dynamics.

Historically, plovers within our metapopulation nested on sandbars within the two rivers, where seasonal water level fluctuations maintained early successional habitat conditions [23, 24]. Before they were altered, the Missouri and Platte rivers experienced peak water levels in March and June due to prairie and mountain snowmelt and precipitation, coinciding with current peak nesting times for plovers [14]. These peak flows submerged existing sandbars and redistributed sediments, creating unvegetated sandbars suitable for plover nesting as water levels receded either within that season or in following seasons [15, 24]. We define a bankfull flow as a high flow event where a river’s water level fills the channel to the top of the river bank and begins to overflow onto the floodplain, while water levels often extend farther into the floodplain during a flood event [25]. Because both bankfull flows and floods are capable of moving sediment and forming or removing sandbars [25], we hereafter combine these terms and refer to them as “high flow events” or “high flows” rather than simply floods. Historically, these rivers experienced more extensive high flow events, approximately once every 4–6 years [24]. Currently, high flow events are less regular and occur approximately every 20 years (based on recent discharge rates from Gavins Point Dam available at http://www.nwd-mr.usace.army.mil/rcc/projdata/gapt.pdf).

The natural hydrographs for these rivers have been altered due to channelization, bank reinforcement, water diversion, and dam construction. Upstream mainstem dam operation and water diversion in the Platte River drainage has significantly altered flow regimes and channel characteristics [26, 27]. Similarly, roughly 35 % of the Missouri River has been impounded in lake ecosystems, and an additional 32 % of the river has been channelized [28]. Dams and reservoirs reduce high flow volumes needed to scour vegetation from existing sandbars and carry sediments downstream [24]. The decline in sediment volume and decreased high flow frequency has decreased the amount of sandbar habitat on parts of the Missouri River by 96 % from 1892 to 2006 [29]. This lack of habitat is now the primary threat to the persistence of plovers in the Great Plains [15].

Today, the majority of adults (77 %; MBB, LRD, JGJ, unpublished data) in the lower Platte River subpopulation (hereafter ‘HC,’ for human created) nest off-river on human-created sand and gravel mines and lakeshore housing developments adjacent to the lower Platte River and a small portion (60 km) of the Loup River [17]. Eighty-one percent of the birds nesting on HC were clustered in the southeastern 1/3 of the study area (MBB, LRD, JGJ, unpublished data; Fig. 1).

The Gavins Point Reach subpopulation occurs in one of the last free-flowing portions of the Missouri River and extends 95 km downstream from the Gavins Point Dam (42° 51′N, 97°29′W). The Lewis and Clark Lake subpopulation is within a reservoir impounded by the dam that contains nesting sandbars at the upstream end (42° 51′N, 97°47′W). From 2005 to 2010, the U.S. Army Corps of Engineers (USACE) built sandbars at both locations to provide nesting habitat for plovers and interior least terns (*Sternula antillarum athalassos*; [16]). Sandbar nesting habitat used by birds on the Gavins Point Reach and Lewis and Clark Lake was generally composed of low, unvegetated mud and sandflats with higher elevation areas of either barren sand or vegetation dominated by cottonwood (*Populus* spp.) and willow (*Salix* spp.) saplings.

In June 2010, unusually large volumes of water were released from Gavins Point Dam, which flooded all active nests and hatched chicks on Gavins Point Reach (hereafter ‘M1F,’ Missouri River site with high flow), reducing reproductive output at this site to 0. In 2011, increased mountain snowpack and spring precipitation resulted in historically high water levels in the Missouri River, and water levels covered all nesting habitat. As a result, few nests were initiated, no nests hatched, and reproductive output at the site was 0 for a second consecutive year.

The two high-water years also created an abundance of nesting habitat for the M1F subpopulation downstream from the dam for the 2012 and 2013 breeding seasons (USACE, unpublished data). The Lewis and Clark Lake subpopulation was largely unaffected by the water level fluctuations (hereafter ‘M2,’ Missouri River site without high flow effects), although some new sandbars were created in 2012 and 2013 from the high velocity of the water passing through the marsh at the upstream end of the lake (USACE, unpublished data). The HC subpopulation was completely unaffected by these water fluctuations (MBB, pers. obs.).

### Field methods

We searched nesting areas at each subpopulation during the plover breeding season (April–August) from 2008 to 2013. At all sites, we visually scanned the area for both banded and unbanded adults and chicks, watched for behaviors suggestive of nesting or breeding, and searched for nests by walking through all potential nesting habitat (i.e., unvegetated and sparsely vegetated wet and dry sand habitat). We captured unbanded, incubating adult (age ≥ 1 yo) plovers using drop door traps placed over their nests, and juvenile (age 0–1 yo) birds were caught by hand as soon after hatching as possible. Once captured, birds were banded using a color band combination that was unique to the individual. We associated adults with nests if the bird was captured on a specific nest or if we observed them incubating eggs or brooding chicks.

Each individual was assigned to one of the subpopulations (HC, M1F, M2) for a given year based on sightings and information about nesting status. If an individual was sighted in multiple subpopulations within the same year, we first assigned birds to a subpopulation based on known breeding locations, or, if that information was unavailable, we assumed that the bird belonged to the subpopulation within which it was most frequently sighted in that year. If there were an equal number of sightings in multiple subpopulations, we ignored the sightings and assigned the bird a value of ‘0’ for ‘unseen in that year’ (only 11 of > 15,000 occasions). All juvenile birds were assigned to the subpopulation in which they hatched.

### Survival and dispersal

To determine what factors affected plover survival and dispersal among our subpopulations, we analyzed capture histories for both juvenile and adult birds from 2008 to 2013 using multi-state mark-recapture models in Program MARK [30]. Multi-state models allow for simultaneous estimation of apparent annual survival (φ), resight (p), and transition (ψ) rates for multiple ‘states’ or ‘strata’ [31, 32]. In our model, the states or strata were the subpopulations of our metapopulation, and transition rates represented the probability of dispersing among the subpopulations. We examined the effect of age class (adult vs. juvenile), year, subpopulation, hatch date, age at banding (in days), reproductive success, distance, and high flow on survival, transition, and resight rates (Additional file 1).

We used the median ĉ test in Program MARK to assess the goodness of fit of the general multi-state model. We estimated overdispersion (ĉ) using the most complex model, such that survival, resight, and transition rates all varied by age class, subpopulation (‘sub’), and year (i.e., age class × sub × year). For all models in this analysis, we fixed juvenile survival and emigration from M1F in 2010 and 2011 at 0 because no fledged chicks were produced during those years at M1F due to high water levels. We used ĉ to adjust standard errors and deviance estimates to account for overdispersion. We used Akaike’s Information Criterion (QAIC_c_; corrected for small sample bias and overdispersion) to rank and to interpret our models [33].

We used a sequential approach for model selection to reduce the number of models under consideration [34] and to create a sufficiently predictive, general model against which we could test our ecological hypotheses about survival and dispersal among the subpopulations in the final step. At each subsequent step, we retained the model structure for parameters in the best model (lowest QAIC_c_) for use in the next step of model selection (Additional file 1).

In the first step of modeling, we varied the model for resight rate (p) while holding survival (φ) and transition models (ψ) at their most complex (i.e., age class × sub × year). In addition, we added individual covariates for the effects of hatch date (‘hatch’) and the age at banding (in days, ‘age’) to all models for survival. We added these covariates to control for known sources of variation in our data [35–37]. Because of a difference in search frequency at each of the subpopulations, all models for resight rate controlled for differences among the subpopulations. We tested all possible combinations (both additive and multiplicative) of age class, year, and subpopulation (8 models; Additional file 1).

In the second step of the model selection procedure, we varied survival rate (φ) while holding transition rate at its most complex structure (i.e., age class × sub × year), and modeled resight rate as the best model (lowest QAIC_c_) from the first step. We assumed *a priori* that juvenile survival would be different than adult survival [35] and controlled for this difference as well as the effects of hatch date and age at banding in all of our models. We tested all possible combinations (both additive and multiplicative) of age class, year, and subpopulation (8 models; Additional file 1).

In the third step of model selection, we varied the model for transition rate while modeling resight and survival rates as the best model from the first and second steps, respectively. We tested all possible combinations (both additive and multiplicative) of age class, year, and subpopulation, including a constant model (15 models; Additional file 1).

In the final step, we tested several hypotheses about survival and transition rates in relation to high flow (habitat availability), distance-mediated dispersal, and reproductive success. Because ‘subpopulation’ and ‘year’ are not necessarily biologically informative parameters, we hypothesized that yearly and subpopulation-specific variation in transition rates could be explained by biologically relevant parameters. For instance, reproductive success can be an important driver of breeding dispersal in many birds [38–40] and has been shown to affect plovers in particular [20]. There is also some evidence that habitat availability (related to flooding of breeding sites) may affect plover dispersal [20]. In addition, dispersal probabilities tend to decrease with increasing distance from a source, although the form of that decrease is often species-specific [39]. We hypothesized that the distance among our sites would be negatively correlated with transition rates. We also hypothesized that site-specific annual reproductive success (average number of chicks fledged/pair at a site where reproductive success in year *t* affects transition rates between years *t* and *t +* 1; [15; MBB, KLH, unpublished data]) and changes in habitat availability would be more parsimonious descriptors of transition probabilities than year-specific and year-by-site specific transition rates.

To model these effects, we used each of the model structures for transition rate from the preceding step within Δ QAIC_c_ < 4 “points” of the top-ranked model. For each of these models, we replaced any occurrence of ‘subpopulation’ with the distance between each site. We replaced any occurrence of ‘year’ with reproductive success, variables for flow (see below), or both flow and reproductive success. Given the observed effects of high flows on M1F (i.e., loss of nesting habitat in 2011, increase in habitat in 2012 in the year after; [15]) and the lack of high flow effects observed for M2 and HC during the study period, we hypothesized that high flows would cause an immediate increase in dispersal out of M1F and a decrease in dispersal into M1F. In addition, we hypothesized the transition rates into M1F from elsewhere would increase for the year(s) following a high flow event as habitat quality and quantity increased.

To test these hypotheses, we created three variables. The first variable for high flow (‘high flow emigration’) affected transition from M1F to the other subpopulations from 2010 to 2011, and the second, high flow variable (‘high flow immigration’) affected transition from M2 or HC to M1F from 2010 to 2011. The variable for ‘post-high flow immigration’ affected any transition from HC or M2 to M1F (where new habitat was created) from 2011 to 2012. All three variables were used simultaneously in modeling transition rates. If a model contained a parameter for age class, we included the interaction between the reproductive success variable and age class under the assumption that juvenile and adult birds would respond differently to reproductive success.

In addition to replacing year and subpopulation for transition rate in this step, we added parameters for reproductive success and high flow to the top survival models to explore the potential effects of these factors on survival. These parameters did not replace subpopulation or year in survival models. We analyzed all model structure combinations for survival and transition rates (32 models; Additional file 1).

All real estimates were model-averaged over all models using QAIC_c_ weights [33]. We used model ranking, *t*-statistics (β/SE), and 95 % confidence limits to interpret the relative size of individual effects (estimated βs) from top-ranked models.

### Metapopulation viability

We used the demographic and transition rates observed in this study to parameterize a population viability analysis (PVA) model for the plover metapopulation formed by the HC, M2, and M1F subpopulations. We used this PVA model to investigate long-term metapopulation viability given the current metapopulation structure, plover demographic rates, and local disturbance regime. The PVA model was constructed in *Vortex* (version 10.0.7.3; [41]), a widely used, previously validated [42] program that simulates the effects of both deterministic forces and demographic, environmental, and genetic stochastic events to assess extinction risk.

**Fig. 2:**
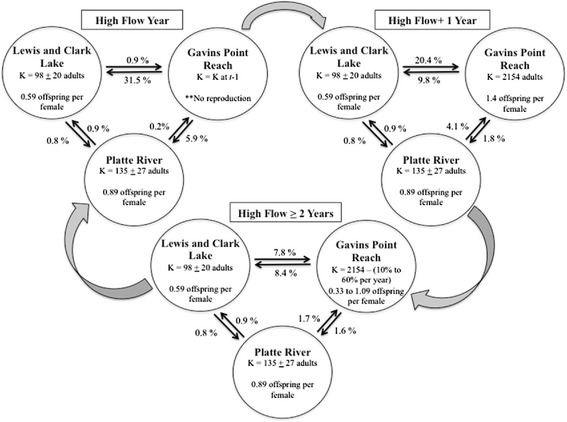
Parameters used in the baseline population viability analysis (PVA) model. All parameters were based on observations described in this study. The large block arrows represent transition between flow and year states. The model operates such that only the demographic rates associated with “High Flow Year” are considered in the year that a high flow event occurs in the model (5 % annual probability). The model then only considers rates associated with “High Flow + 1 Year” in the year immediately following a high flow event and rates associated with “High Flow ≥ 2 Years” for all other years until the next high flow event occurs. Values above black arrows indicate the percentage of adults that disperse from a population into the adjoining population, and “K” indicates the carrying capacity of habitat available to the population

**Fig. 3:**
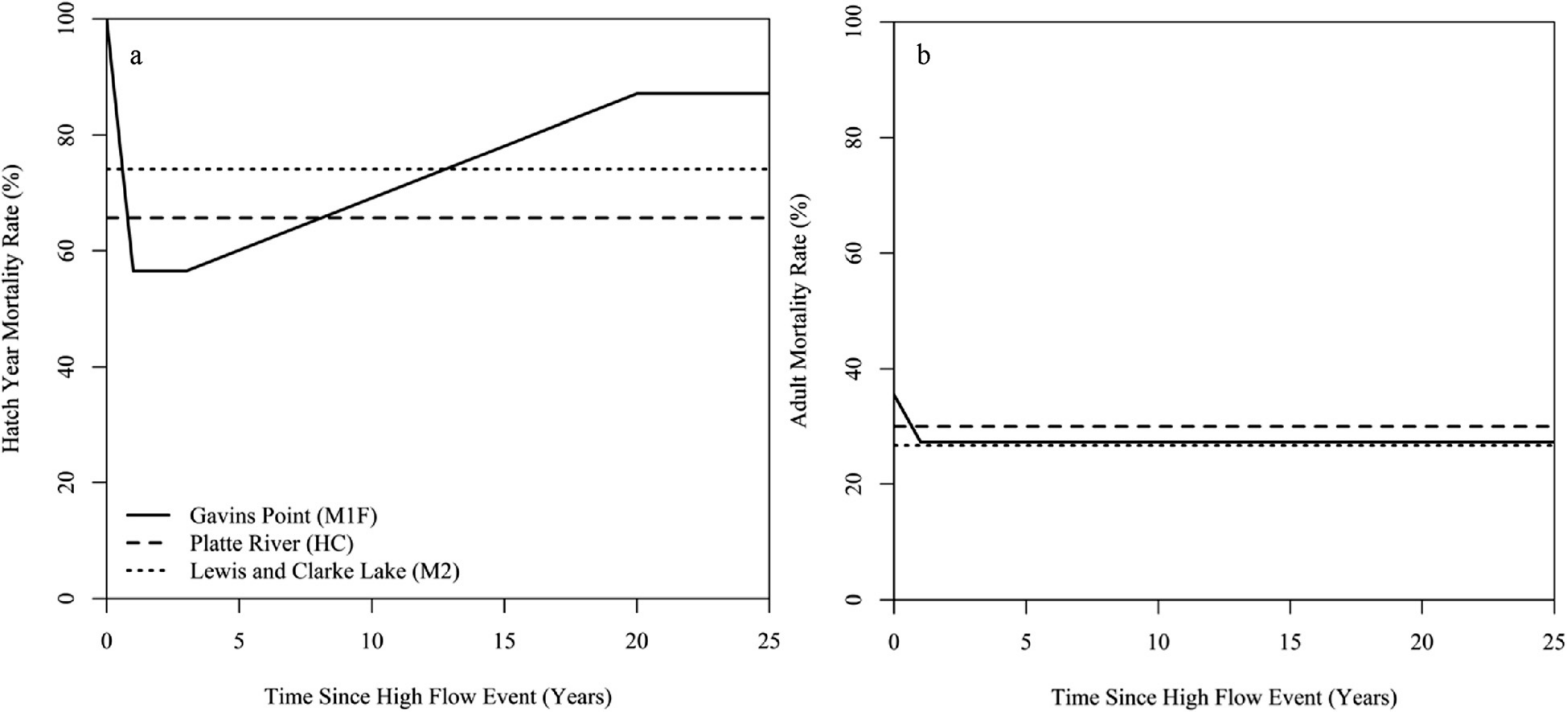
Piping plover mortality rates used in the baseline population viability analysis (PVA) model. Mortality rates were calculated for (**a**) hatch years and (**b**) adults and were specific to each population in the metapopulation (Gavins Point Reach , M1F; Platte River, HC; and Lewis and Clarke Lake, M2). Rates were a function of the time since the last high flow event occurred for the M1F population only

Transition and demographic rates were specific to each subpopulation, and many of these rates were dependent on the time since a high flow event last occurred (Fig. 2; Fig. 3). High flow events occurred stochastically in the model with a frequency of 5 % (i.e., 1 high flow event approximately every 20 years) only for the M1F subpopulation. See Additional file 2 for all parameters and functions used in *Vortex*. We simulated the metapopulation model for 1000 stochastic replicates of 100 years to estimate mean values for extinction risk, population size, and time to extinction.

**Fig. 4:**
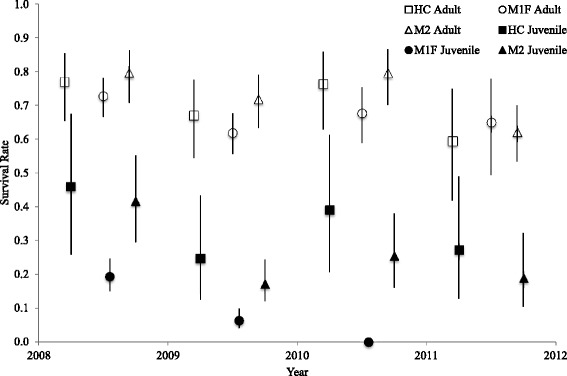
Survival rate of adult (1+ yo; unfilled) and juvenile (0–1 yo; filled) piping plovers from 2008–2012. Survival rates were separated by population: lower Platte River (squares), the Gavins Point Reach of the Missouri River (circles), and Lewis and Clark Lake (triangles). High flow events occurred in June of 2010 and May of 2011. No chicks were produced on M1F in 2011 because of flooding. Error bars represent 95 % confidence limits

We did not make demographic rates or carrying capacity for HC and M2 subpopulations dependent on the occurrence of high flow events in the PVA model in accordance with the observations made as part of this study. However, following our results, we made immigration rates into these subpopulations from M1F and emigration rates from these subpopulations into M1F dependent on high flows (Fig. 2). We also made demographic and transition rates for M1F a function of high flows in the PVA model given observations made as part of this study. In the model, mortality and emigration increased, immigration rates decreased, and reproduction declined to zero for this subpopulation during a high flow year. In the year after a high flow event (i.e., the “high flow +1 year”), the amount of newly created suitable habitat increased, increasing the subpopulation’s carrying capacity (see Additional file 2 for details on the calculation of carrying capacity), drawing an increased number of immigrants from other subpopulations, and decreasing mortality rates either to baseline levels (adults) or to the lowest observed levels (juveniles). After the high flow + 1 year, we assumed that immigration/emigration rates would stabilize at baseline levels and that mortality rates would increase annually until the next high flow event occurred following a 3-year window of low mortality for juveniles (KLH unpublished data; Fig. 2; Fig. 4a). In addition, we allowed the amount of suitable habitat to decline each year in M1F by 10–60 % based on empirical data collected previously on M1F [15] until the next high flow event, simulating the impact of erosion and vegetation encroachment (Additional file 2). Finally, we assumed that the standard deviation due to environmental variation for mortality rates was equivalent to 20 % of the mortality rate in a non-high flow, baseline year for all three subpopulations.

## Results

We banded and encountered 2640 plovers from 2008–2013 across all sites. Of these, 93 adults and 245 juveniles were banded or first encountered within the HC subpopulation, 470 adults and 1117 juveniles were banded/encountered within the M1F subpopulation, and 212 adults and 503 juveniles were banded/encountered within the M2 subpopulation. Chicks within HC hatched on 14 Jun ± 14 days (mean ± SD, range: 15 May–26 Jul) and were 5 ± 6 days old at banding (mean ± SD, range: 0–24). Chicks within M1F hatched on 30 Jun ± 15 days (mean ± SD, range: 26 May–3 Aug) and were 1 ± 2 days old at banding (mean ± SD, range: 0–16). Chicks within M2 hatched on 3 Jul ± 12 days (mean ± SD, range: 6 Jun– 3 Aug) and were 2 ± 3 days old at banding (mean ± SD, range: 0–24). Mean reproductive output from 2008–2012 was highest within M2, followed by HC and then M1F (Table 1).

**Table 1:** Estimate of fledged piping plover chicks produced/pair^a^ from 2008–2012

	Subpopulation		
Breeding season	HC	M1F	M2
2008	1.20	1.14	1.56
2009	0.61	0.53	2.61
2010	0.74	0.00^b^	1.87
2011	1.31	0.00^b^	0.49
2012	1.44	1.80	1.27
Mean	1.06	0.69	1.56

Estimates were separated by subpopulation: the lower Platte River (HC), the Gavins Point Reach of the Missouri River (M1F), and Lewis and Clark Lake (M2). These values were used to predict survival and transition rates in a multistate mark recapture model

^a^ We estimated chicks fledged/pair as follows: Clutch size (3.73; DHC, unpublished data) × Female Success (Probability that a female has a successful nest. This value was used to account for repeated nesting following failures) × Chick survival to fledge. These values were estimated using nest survival and chick survival estimates from each of the subpopulations

^b^ High flows reduced reproductive output to 0 on M1F in 2010 and 2011

Resighting rates of banded birds varied by age class, subpopulation, and year. Rates were highest at M1F (adult: 0.92 ± 0.06, juvenile: 0.74 ± 0.13; mean ± SD) and M2 (adult: 0.88 ± 0.08, juvenile: 0.66 ± 0.14; mean ± SD) relative to those on HC (adult: 0.64 ± 0.14, juvenile: 0.32 ± 0.11; mean ± SD).

### Survival

Plover survival rates varied by age class, subpopulation, and year (Table 2; Fig. 4). Regardless of subpopulation, juvenile survival (0.24 ± 0.14, mean ± SD) was lower than adult survival (0.70 ± 0.07, mean ± SD; Fig. 4). Both adult and juvenile birds tended to have higher annual survival within HC (adult: 0.70 ± 0.07, juvenile: 0.34 ± 0.08, mean ± SD) and M2 (adult: 0.73 ± 0.07, juvenile: 0.26 ± 0.10, mean ± SD) compared to plovers within M1F (adult: 0.67 ± 0.04, juvenile: 0.09 ± 0.08, mean ± SD; Fig. 4).

**Table 2:** Multistate mark recapture model ranking for piping plover survival and transition from 2008–2012

Model	k	ΔQAIC_c_^a^	Deviance	QAIC_c_ weight
φ _age×sub + age×year + juvenile:(band + hatch) + high flow_ ψ _age + distance + high flow_^b^	31	0.000	4525.731	0.286
φ _age×sub + age×year + juvenile:(band + hatch) + high flow_ ψ _age×success + distance + high flow_	33	0.738	4522.393	0.197
φ _age×sub + age×year + juvenile:(band + hatch) + high flow + adult:success_ ψ _age + distance + high flow_	32	1.993	4525.687	0.105
φ _age×sub + age×year + juvenile:(band + hatch)_ ψ _age + distance + high flow_	29	1.996	4531.798	0.105
φ _age×sub + age×year + juvenile:(band + hatch) + adult:success_ ψ _age + distance + high flow_	30	2.308	4530.075	0.090
φ _age×sub + age×year + juvenile:(band + hatch)_ ψ _age×success + distance + high flow_	31	2.675	4528.406	0.075
φ _age×sub + age×year + juvenile:(band + hatch) + high flow + adult:success_ ψ _age×success + distance + high flow_	34	2.731	4522.346	0.073
φ _age×sub + age×year + juvenile:(band + hatch) + adult:success_ ψ _age×success + distance + high flow_	32	2.996	4526.690	0.064

Only top-ranked models (ΔQAIC_c_ ≤ 4) are shown. Resight rate ‘p’ did not differ among the top-ranked models – p _age + sub + year_

^a^Minimum QAIC_c_ = 4588.307

^b^Subscripts represent the covariates that affected apparent survival (φ) and transition (ψ) rates. Age – juvenile (0–1 year post-hatch) and adult (1+ years post-hatch) rates differ (a ‘:’ indicates that the covariate(s) affect only that age-class); sub – survival and transition rates differ by subpopulation; year – survival and transition rates differ by year; band – age at banding (in days, affected juvenile birds only and appeared in all models) effect on survival; hatch – hatch date (in days, affected juvenile birds only and appeared in all models) effect on survival; high flow (survival) – 2 variables for the effects of (*i*) the high flow events on M1F survival (from 2010–2011) and (*ii*) the immediate post-high flow environment on M1F survival (from 2011–2012); high flow (transition) – 3 variables for the effects of (*i*) high flow on emigration from M1F to the other areas (from 2010–2011), (*ii*) high flow on immigration into M1F from the other areas (from 2010–2011), and (*iii*) the post-high flow environment on the immigration of individuals into M1F from the other areas (from 2011–2012); success – the effect site-specific reproductive output (chicks fledged/pair) on survival and transition (affected only adult birds for survival); and distance – the effect of the distance among subpopulations on transition rates

Survival of adult birds in M1F was higher compared to survival in M2 and HC following the high flows but was lower before and during the event (Fig. 4). Survival in all subpopulations from 2011–2012 was relatively low (Fig. 4). Variables for flow appeared in the top three models (Table 2), and point estimates suggested that high flows were correlated with a negative effect on survival during the event but a positive effect in the year following (Table 3). The 95 % CI and magnitude of the β/SE values indicated stronger support for the post-high flow effect than an effect of flow on survival, which was not significant (Table 3).

**Table 3:** Beta coefficients of the effect of group- and individual-specific covariates on survival and transition parameters from the top-ranked multi-state mark recapture model

Parameter	Covariate^a^	Estimate	SE	|β/SE|^b^	Lower 95 % CL	Upper 95 % CL
Survival (φ)	High flow	−0.276	0.377	0.732	−1.016	0.464
	Post-high flow	0.785	0.382	2.055	0.036	1.534
	Hatch Date (juvenile)	−0.031	0.008	3.875	−0.047	−0.016
	Age at Banding (juvenile)	0.032	0.035	0.914	−0.037	0.101
Transition (ψ)	Distance	−0.011	0.001	11.000	−0.014	−0.008
	High flow Emigration	1.690	0.256	6.602	1.189	2.191
	High flow Immigration	−2.279	1.026	2.221	−4.289	−0.268
	Post-high flow Immigration	1.010	0.251	4.024	0.517	1.502

^a^Covariates used to predict survival and transition rates included: reproductive success – the effect of the number of chicks fledged/pair calculated for each subpopulation on both survival and transition rates; ‘high flow’ for the effect of high flows on survival of birds on M1F during the high flow ever (2010–2011); ‘post-high flow’ for the effect of the immediate post-high flow environment on survival on M1F (2011–2012); ‘hatch date’ controlled for this effect on hatch-year survival (appeared in all models); ‘age at banding’ controlled for this effect on hatch-year survival (appeared in all models); ‘distance’ for the effect of linear distances among different breeding areas on transition probabilities; ‘high flow emigration’ for the effect of high flows on emigration from M1F to the other subpopulations (2010–2011); ‘high flow immigration’ for the effect of high flows on immigration into M1F from other subpopulations (2010–2011); ‘post-high flow immigration’ for the effect of the immediate post-high flow environment on the immigration of individuals into M1F from the other subpopulations; and juvenile and adult indicate age-specific estimates

^b^The absolute value of the estimate divided by its standard error (i.e., *t*-value). This value allows comparison among the estimates scaled by standard error

There was little indication from model ranking that reproductive success affected survival (Table 2). The highest ranked model that included reproductive success had a weight of only 0.105 (Table 2), and the confidence limits for the coefficient included 0, indicating that the effect was not precisely estimated (Table 3). Birds that hatched later in the year had lower survival than those hatching earlier. Age at banding had a positive effect on juvenile survival, but the 95 % CI and magnitude of the β/SE value for age at banding indicated that this variable had only a marginal effect relative to the other covariates in the top-ranked model (Table 3).

### Dispersal

Biologically relevant parameters (i.e., age-class, distance between sites, and flooding) were more parsimonious (lower QAIC_c_ values) descriptors of plover dispersal than the subpopulation and year parameters (Table 2). Neither subpopulation nor year appeared in the competing (QAIC_c_ ≤ 4) transition rate models. The highest ranked models containing subpopulation were > 10 QAIC_c_ “units” from the top-ranked model and had essentially 0 weight. The highest ranked model containing year was > 32 QAIC_c_ points from the top-ranked model.

**Fig. 5:**
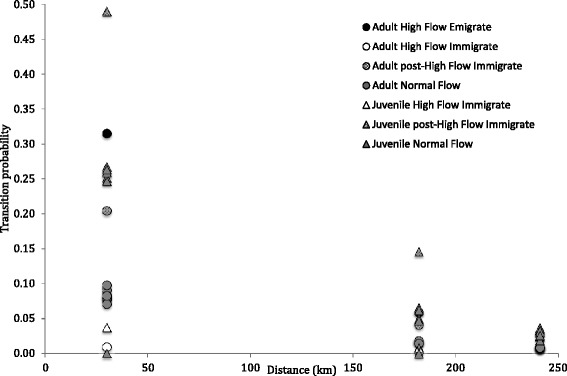
Model- averaged transition rates of adult and juvenile piping plovers moving among subpopulations on the lower Platte River (HC), the Gavins Point Reach (M1F) of the Missouri River, and Lewis and Clark Lake (M2) from 2008–2012

Juvenile birds had higher average transition rates (0.11 ± 0.12) than adult birds (0.05 ± 0.07, mean ± SD; Fig. 5). Plover transition rates decreased with increasing distance (Table 3; Fig. 5), and emigration and immigration rates were affected by high flows and the creation of habitat thereafter (Table 3). The covariates for high flow and distance appeared in all competing models (Table 2), and the β/SE values indicated that the strongest effect on transition rate was from distance, followed by emigration during the high flow event and then immigration after the event (Table 3).

High flows increased transitions from M1F (‘high flow emigrate’) and decreased transitions into M1F (‘high flow immigrate’), and the post-high flow environment was associated with increased immigration rates into M1F (‘post-high flow immigrate’; Table 3). The inclusion of reproductive output in the transition rate model did not substantially improve fit (top-ranked model vs. second ranked model; Table 2). The interaction between reproductive success and age class appeared in the second-ranked model but did not decrease the deviance enough to outweigh the penalty (Table 2).

### Population viability

**Fig. 6:**
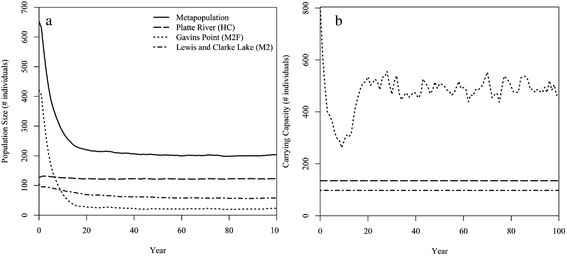
**a** Subpopulation sizes and **b** carrying capacities for a piping plover metapopulation. The metapopulation was composed of populations on the lower Platte River (HC) and on the Missouri River at Gavins Point Reach (M1F) and Lewis and Clarke Lake (M2). Population trends were simulated for 1000 replicates of 100 years in the population viability analysis program *Vortex*

Results of the PVA model showed that the metapopulation has a low probability of extinction at 0.0 % and an average population size of 203 adults after 100 years (Table 4; Fig. 6a). HC and M2 were also persistent with low extinction probabilities (0.0 % and 0.3 %, respectively) and subpopulation sizes of 123 and 58 adults, respectively, after 100 years (Table 4; Fig. 6a). The M1F subpopulation had by far the highest risk of extinction at 47.7 %. In addition, although the average carrying capacity at M1F over a 100-year simulation was 477 adults, the subpopulation size rarely approached this level, supporting only 22 adults by year 100 (Table 4; Fig. 6b). In replicate iterations where local extinction did occur, M1F tended to become extinct by year 21 on average, but it was often recolonized by dispersers from the other subpopulations (2593 recolonizations occurred in 1000 replicates of 100 years).

**Table 4:** Extinction risk for the piping plover metapopulation

	Extinction probability (SE)	Mean years to extinction (SE)	Mean subopulation size (individuals)			λ_deter_^a^	λ_stoch_^b^
			20 years	50 years	100 years		
Metapopulation	0.0 % (0.0)	NA	220	212	203	NA	1.1
HC	0.0 % (0.0)	NA	123	121	123	1.1	1.1
M1F	47.7 % (1.6)	21.3 (0.4)	27	21	22	0.9	1.1
M2	0.5 % (0.2)	65.3 (2.7)	69	60	58	1.0	1.0

The metapopulation was composed of subpopulations on the lower Platte River (HC) and on the Missouri River at Gavins Point Reach (M1F) and Lewis and Clarke Lake (M2). Trends were simulated for 1000 stochastic replicates of 100 years in the population viability analysis program *Vortex*

^a^Finite rate of population increase, deterministic

^b^Finite rate of population increase, stochastic

The overall low risk of metapopulation extinction was due largely to the persistence of HC. Adults in HC comprised 60.6 % of the total metapopulation by year 100, compared to only 11.0 % from M1F despite the fact that this subpopulation had a substantially larger carrying capacity in most years (Table 4; Fig. 6b). Furthermore, M1F had a deterministic finite rate of increase (λ_deter_) of 0.9, indicating that, based on a life table analysis of the mean mortality and reproductive rates initially used to parameterize the model, the population should decline. However, the stochastic finite rate of population increase (λ_stoch_), which also takes into account stochastic fluctuations and immigration/emigration throughout the course of a simulation, was 1.1 (Table 4). This result indicates that M2 and HC supported the persistence of M1F given the frequency of high flow events that currently occurs along the Missouri River.

## Discussion

The metapopulation dynamics in this study were strongly influenced by landscape dynamics, with functional connectivity (as measured through dispersal rates) changing through time as a result of both natural (high flows) and anthropogenic (isolation by distance) factors. It is clear that extreme events, including natural disturbances, can profoundly influence a species’ evolutionary history and population dynamics [6, 43, 44]. Large floods and bankfull flows historically occurred regularly on the Missouri River and its tributaries and can exert a primary source of selective pressure for adaptation as a cause of mortality in species like piping plovers [45]. Such species can exhibit life history adaptations (e.g., synchronization of life history events in relation to a common flow regime) and/or behavioral adaptations (e.g., adaptations that allow a species to respond directly to high flow events) in response to the magnitude, frequency, seasonal timing, predictability, and/or duration of these events [45]. This study and others have identified several such potential adaptations in plovers in the Great Plains. For instance, before the prevalence of dams, the Missouri River and surrounding tributaries experienced relatively predictable, high water flow peaks in March and late May/early June [24]. Plovers in this region typically begin laying eggs in mid- to late May, which, in addition to avoiding much of the inclement weather in the spring and producing chicks near peak invertebrate abundance in mid- to late-summer, allowed them to produce offspring after waters had receded to expose high quality habitat [23]. Plovers may also exhibit behavioral adaptations that allow them to tolerate high flows, such as their ability to renest up to four times in a breeding season in the event that eggs or young hatchlings are lost to flooding or predation [46]. Such life history adaptations are expected for species in dynamic environments where disturbances are frequent, large, and predictable [45], characteristic of the historical Missouri River [24].

Our results also indicate that plovers increase their dispersal rates and reproductive output following high flow events (or large-scale fluctuations in the amount of habitat). Species in naturally disturbed environments generally display compensatory or stabilizing effects to counterbalance higher mortality during a disturbance [47]. In our study, adult mortality increased slightly while hatch-year mortality was 100 % within M1F as a result of high flows, and identical trends have been observed in a piping plover population on the Platte River [48] as well as in other waterbird species [49]. At M1F, higher flow-related mortality in 2010–2011 was compensated for in 2012 and 2013 by lower mortality and higher reproductive output, likely due to density-dependent increases in chick survival (KLH, unpublished data) and nesting [50] related to high flows in this system [48]. Higher recruitment following high flow-related mortality has also been demonstrated in crimson finches (*Neochmia phaeton*; [51]) and some Australian waterbirds [49]. Therefore, these compensatory mechanisms (i.e., low mortality and high reproductive output following high flows) could be important adaptations in plovers and other riverine species that promote population persistence in response to high flows, which would have historically occurred multiple times within a plover’s lifespan in the Great Plains [24].

Piping plovers generally exhibit high site fidelity [52], which could benefit a species in somewhat unpredictable, dynamic environments [53]. Our observations in normal flow years, showing lower dispersal rates between all pairs of populations, further support the propensity for site fidelity in this species. In contrast, we found dispersal rates from M1F increased substantially during the 2010–2011 high flow years as some birds left inundated habitat and moved to the HC and M2 populations, while dispersal rates from M2 and HC into M1F increased significantly after 2011 when high quality habitat was created. High flows and other extreme events appear to be important dispersal cues for many otherwise site faithful species, including several birds and fish [44, 49, 54–57]. Increased dispersal rates into M1F were critical to the persistence and recolonization of this subpopulation. Therefore, this study confirms that natural disturbance is a strong driver of dispersal in this system, and disturbance-related shifts in functional connectivity had a major influence on metapopulation dynamics and the persistence of individual subpopulations.

Although high flows clearly influenced plover dispersal rates, the precise cues used by this species to make dispersal decisions remain unclear. Individuals could have responded to some regional abiotic cue associated with the high flow event itself ([57]). Alternatively, individuals could have reacted directly to the amount of nesting habitat or to the lower population size within M1F after the event; immigration rates in plovers and other birds can be density dependent [14, 39, 58]. Dispersal in plovers was previously linked to reproductive success, exhibiting a positive relationship between emigration rates and nest failure at a given site [20]. The relationship between individual reproductive success and dispersal is well established in birds [38–40], and there is growing evidence that individuals use information on local, conspecific reproductive success to make dispersal decisions [58–61]. Although the evidence that reproductive output within a location was correlated with dispersal in this study was weak, increased emigration out of M1F following total reproductive failure could be related to reproductive success as well as habitat loss. Irrespective of the root cause, high flows along the Missouri River were closely related to increased functional connectivity in this plover metapopulation.

If plovers have adapted certain life history attributes and behaviors to the natural flow regime, relatively recent alterations to this disturbance regime may have far-reaching effects on the species’ population dynamics and viability [3, 45]. Dams along the Missouri River and its tributaries have almost completely eliminated high flows, and only the most catastrophic events (e.g., the 2010–2011 event observed in this study) have the capacity to exceed dam storage capabilities [24]. In the absence of habitat-creating flows, we would expect productivity and dispersal rates to remain at low levels for extended periods of time, consistent with rates observed prior to the 2010–2011 high flow events [14]. Furthermore, nearly 70 % of the Missouri River is either impounded or channelized [28], which has increased the distance among areas with suitable habitat. Plovers will use reservoir shorelines to nest, but many of the reservoirs do not have shorelines suitable for nesting [62]. If connectivity is depressed in the absence of flow-based cues or because of increased isolation from channelization and impoundments, the benefits of dispersal may also decline for this plover metapopulation. The spatial structure and connectivity patterns within metapopulations can govern critical genetic and demographic processes [3]. Decreased connectivity could reduce the likelihood of the recolonization of an extirpated population [63] or eliminate demographic rescue effects for a declining population [64]. A reduction in dispersal also could increase the risks of a population experiencing inbreeding depression or genetic drift [65], particularly given the small sizes of these plover subpopulations. Therefore, a loss of flow-based dispersal cues could have important implications for this, and other, piping plover metapopulations.

The implications of the loss of high flows for this species, which has various adaptations to a natural flow regime, can be seen in the results of the PVA and a related study that investigated multiple management and flow scenarios [66]. In this study, M1F had a relatively high extinction risk (47.7 %) under the current flow regime and was predicted to support only a very small total population (22 adults). Twenty-two adults is exactly the number observed in 1996 and 1997 at M1F, the nadir during current monitoring and before a substantial high water event. These rates translated to a total predicted average metapopulation size of approximately 200 adults, far below the average expected carrying capacity of 700 adults. In contrast, separate simulations predicted metapopulation size would be substantially higher under a more frequent flow regime, reminiscent of a flow regime to which plovers in the Great Plains were originally adapted [66].

### Conclusions

Conservation-reliant species require some level of management to prevent extinction or extirpation [67]. Piping plovers will likely require varying levels of habitat management, beach closure, predator control, monitoring, and captive rearing throughout their range to maintain and to grow populations [15, 68]. In our study, the predicted persistence of the HC and M2 populations is dependent upon the assumption of consistent management and availability of nesting habitat at these sites to maintain the carrying capacity and reproductive output. This result may have been related to the lack of correlation of disturbances among subpopulations; increased autocorrelation in habitat turnover can negatively impact metapopulation persistence [3].

Human-created sites that support HC and M2 are critical to the survival of the metapopulation, by providing both stable habitat and dispersers. Although habitats used by these populations were relatively stable during our study period and were modeled as such, habitat was created through very different processes. Habitat used by HC is created by sand and gravel mining operations and residential housing development construction. Mining practices involve mechanical disturbance of dredged sand, which maintains sparsely vegetated expanses of sand adjacent to water [15]. The conversion of mines to housing developments requires additional mechanical disturbance to redistribute the sand so that the topography is suitable for housing construction.

Sandbars used by plovers within M2 during this study were created by the USACE in order to comply with requirements of the Endangered Species Act [16, 35]. These human-created sandbars require vegetation management, sand augmentation, and predator control [16] to maintain their usefulness beyond a few years. Management of human-created HC habitat is based on agreements between private interests (mining companies or real estate developers) and regulatory agencies and is limited mostly to implementing measures that avoid “take”. Thus, while the USACE is incentivized to create and maintain habitat in M2 to meet regulatory obligations, private interests in the areas used by HC are not [17]. Consequently, evolving mining practices and economic conditions could appreciably alter the amount, configuration and distribution of habitat used at HC.

Our analysis of this controlled, natural experiment (albeit with low replication) indicated that despite a low overall probability of metapopulation extinction, the persistence was predicated on temporarily stable, anthropogenically modified habitats as well as dispersal among these subpopulations. Given the highly dynamic nature of habitat availability in this system, maintaining several populations within the metapopulation, stable sources of habitat, and assuring connectivity through dispersal may be critical.

## Additional files

Additional file 1:
**Stepwise modeling of apparent survival (φ), resight rate (p), and transition rate (ψ).** File contains all survival models tested and detailed information about the steps taken to build those models. (DOCX 24 kb) Additional file 2:
**Parameters used in Vortex ver.10 to estimate the population viability of a Great Plains piping plover (** ***Charadrius melodus*** **) metapopulation.** The metapopulation was composed of three populations located (*i*) along the lower Platte River (HC, human created habitat) and in the Missouri River on (*ii*) the Gavins Point Reach (M1F, Missouri River site, high flow) and (*iii*) Lewis and Clark Lake (M2, Missouri River site, no high flow). File contains all parameters used in *Vortex* for population viability analysis modeling. (DOCX 98 kb)
