# Supplementary material for: Metapopulation viability of an endangered shorebird depends on dispersal and human-created habitats: piping plovers (Charadrius melodus) and prairie rivers
Source: Mov Ecol. 2016 Mar 15;4:6. doi: 10.1186/s40462-016-0072-y (PMC4791857; doi:10.1186/s40462-016-0072-y)
Supplement: Additional file 1: — Stepwise modeling of apparent survival (φ), resight rate (p), and transition rate (ψ). File contains all survival models tested and detailed information about the steps taken to build those models. (DOCX 24 kb) [file 40462_2016_72_MOESM1_ESM.docx]

Additional File 1: Stepwise modeling of apparent survival (φ), resight rate (p), and transition rate (ψ). For step 1 we varied the structure for resight rate while holding survival and transition rates the same. In step 2, we used the top-ranked (lowest QAIC_c_) structure for resight rate, and varied the structure for survival, while holding transition constant. In step 3, we used the top-ranked structures from steps 1 and 2 and varied the structure of the transition rate. In the final step, we took any model with a ΔQAIC_c_ ≤ 4.0 and replaced any occurrence of subpopulation with distance and any occurrence of year with reproductive success and/or high flow for transition rates. We also tested for an additive effect of reproductive success and high flows on survival in this step.

| φ | p | ψ | QAIC_c_ | ΔQAIC_c_^a^ |
| --- | --- | --- | --- | --- |
| ***Step 1*** |  |  |  |  |
| age × sub × year + juvenile:(band + hatch)^b^ | age + sub + year | age × sub × year | 4630.65 | 0.00 |
| age × sub × year + juvenile:(band + hatch) | age + sub | age × sub × year | 4632.47 | 1.83 |
| age × sub × year + juvenile:(band + hatch) | sub + age × year | age × sub × year | 4636.22 | 5.57 |
| age × sub × year + juvenile:(band + hatch) | age × sub + age × year | age × sub × year | 4636.63 | 5.98 |
| age × sub × year + juvenile:(band + hatch) | age × sub + year | age × sub × year | 4638.35 | 7.71 |
| age × sub × year + juvenile:(band + hatch) | age × sub × year | age × sub × year | 4639.64 | 8.99 |
| age × sub × year + juvenile:(band + hatch) | age × sub | age × sub × year | 4640.59 | 9.94 |
| age × sub × year + juvenile:(band + hatch) | sub | age × sub × year | 4647.45 | 16.80 |
| ***Step 2*** |  |  |  |  |
| age × sub + age × year + juvenile:(band + hatch) | age + sub + year | age × sub × year | 4622.33 | 0.00 |
| age × sub + year + juvenile:(band + hatch) | age + sub + year | age × sub × year | 4625.07 | 2.74 |
| age + year + sub + juvenile:(band + hatch) | age + sub + year | age × sub × year | 4627.45 | 5.12 |
| age × year + sub + juvenile:(band + hatch) | age + sub + year | age × sub × year | 4628.31 | 5.97 |
| age × sub × year + juvenile:(band + hatch) | age + sub + year | age × sub × year | 4630.65 | 8.31 |
| age × year + juvenile:(band + hatch) | age + sub + year | age × sub × year | 4643.47 | 21.13 |
| age + year + juvenile:(band + hatch) | age + sub + year | age × sub × year | 4645.01 | 22.68 |
| age + juvenile:(band + hatch) | age + sub + year | age × sub × year | 4673.08 | 50.74 |
| ***Step 3*** |  |  |  |  |
| age × sub + age × year + juvenile:(band + hatch) | age + sub + year | age × sub × year | 4622.33 | 0.00 |
| age × sub + age × year + juvenile:(band + hatch) | age + sub + year | sub × year | 4638.53 | 16.20 |
| age × sub + age × year + juvenile:(band + hatch) | age + sub + year | age × year + sub | 4640.69 | 18.36 |
| age × sub + age × year + juvenile:(band + hatch) | age + sub + year | age + year + sub | 4640.82 | 18.49 |
| age × sub + age × year + juvenile:(band + hatch) | age + sub + year | age × sub + year | 4647.75 | 25.41 |
| age × sub + age × year + juvenile:(band + hatch) | age + sub + year | age × sub + age × year | 4647.82 | 25.48 |
| age × sub + age × year + juvenile:(band + hatch) | age + sub + year | age + sub | 4652.97 | 30.63 |
| age × sub + age × year + juvenile:(band + hatch) | age + sub + year | age × sub | 4660.81 | 38.47 |
| age × sub + age × year + juvenile:(band + hatch) | age + sub + year | sub | 4676.28 | 53.94 |
| age × sub + age × year + juvenile:(band + hatch) | age + sub + year | year + sub | 4676.33 | 54.00 |
| age × sub + age × year + juvenile:(band + hatch) | age + sub + year | age × year | 4742.36 | 120.02 |
| age × sub + age × year + juvenile:(band + hatch) | age + sub + year | age + year | 4749.53 | 127.20 |
| age × sub + age × year + juvenile:(band + hatch) | age + sub + year | age | 4753.11 | 130.78 |
| age × sub + age × year + juvenile:(band + hatch) | age + sub + year | null | 4772.85 | 150.52 |
| age × sub + age × year + juvenile:(band + hatch) | age + sub + year | year | 4775.03 | 152.70 |
| ***Step 4*** |  |  |  |  |
| age × sub + age × year + juvenile:(band + hatch) + high flow | age + sub + year | age + distance + high flow | 4588.31 | 0.00 |
| age × sub + age × year + juvenile:(band + hatch) + high flow | age + sub + year | age + distance + high flow + age × success | 4589.05 | 0.74 |
| age × sub + age × year + juvenile:(band + hatch) + high flow+ adult:success | age + sub + year | age + distance + high flow | 4590.30 | 1.99 |
| age × sub + age × year + juvenile:(band + hatch) | age + sub + year | age + distance + high flow | 4590.30 | 2.00 |
| age × sub + age × year + juvenile:(band + hatch) + adult:success | age + sub + year | age + distance + high flow | 4590.61 | 2.31 |
| age × sub + age × year + juvenile:(band + hatch) | age + sub + year | age + distance + high flow + age × success | 4590.98 | 2.68 |
| age × sub + age × year + juvenile:(band + hatch) + high flow+ adult:success | age + sub + year | age + distance + high flow + age × success | 4591.04 | 2.73 |
| age × sub + age × year + juvenile:(band + hatch) + adult:success | age + sub + year | age + distance + high flow + age × success | 4591.30 | 3.00 |
| age × sub + age × year + juvenile:(band + hatch) + high flow | age + sub + year | age × sub + high flow + age × success | 4599.38 | 11.07 |
| age × sub + age × year + juvenile:(band + hatch) + high flow | age + sub + year | age × sub + high flow | 4599.39 | 11.09 |
| age × sub + age × year + juvenile:(band + hatch) | age + sub + year | age × sub + high flow + age × success | 4601.15 | 12.85 |
| age × sub + age × year + juvenile:(band + hatch) | age + sub + year | age × sub + high flow | 4601.17 | 12.87 |
| age × sub + age × year + juvenile:(band + hatch) + high flow+ adult:success | age + sub + year | age × sub + high flow + age × success | 4601.42 | 13.11 |
| age × sub + age × year + juvenile:(band + hatch) + high flow+ adult:success | age + sub + year | age × sub + high flow | 4601.43 | 13.13 |
| age × sub + age × year + juvenile:(band + hatch) + adult:success | age + sub + year | age × sub + high flow + age × success | 4601.82 | 13.51 |
| age × sub + age × year + juvenile:(band + hatch) + adult:success | age + sub + year | age × sub + high flow | 4601.90 | 13.59 |
| age × sub + age × year + juvenile:(band + hatch) + high flow | age + sub + year | age + distance + age × success | 4608.91 | 20.60 |
| age × sub + age × year + juvenile:(band + hatch) + high flow+ adult:success | age + sub + year | age + distance + age × success | 4610.75 | 22.44 |
| age × sub + age × year + juvenile:(band + hatch) + adult:success | age + sub + year | age + distance + age × success | 4610.95 | 22.64 |
| age × sub + age × year + juvenile:(band + hatch) | age + sub + year | age + distance + age × success | 4611.45 | 23.14 |
| age × sub + age × year + juvenile:(band + hatch) + high flow | age + sub + year | age × sub + age × success | 4618.30 | 29.99 |
| age × sub + age × year + juvenile:(band + hatch) + high flow+ adult:success | age + sub + year | age × sub + age × success | 4620.25 | 31.95 |
| age × sub + age × year + juvenile:(band + hatch) | age + sub + year | age × sub + age × success | 4620.53 | 32.22 |
| age × sub + age × year + juvenile:(band + hatch) + adult:success | age + sub + year | age × sub + age × success | 4620.57 | 32.26 |
| age × sub + age × year + juvenile:(band + hatch) + high flow | age + sub + year | age × sub × year | 4620.82 | 32.51 |
| age × sub + age × year + juvenile:(band + hatch) | age + sub + year | age × sub × year | 4622.33 | 34.03 |
| age × sub + age × year + juvenile:(band + hatch) + adult:success | age + sub + year | age × sub × year | 4622.50 | 34.20 |
| age × sub + age × year + juvenile:(band + hatch) + high flow+ adult:success | age + sub + year | age × sub × year | 4622.84 | 34.54 |
| age × sub + age × year + juvenile:(band + hatch) + high flow | age + sub + year | age + distance + age × year | 4641.14 | 52.83 |
| age × sub + age × year + juvenile:(band + hatch) + adult:success | age + sub + year | age + distance + age × year | 4642.95 | 54.64 |
| age × sub + age × year + juvenile:(band + hatch) + high flow+ adult:success | age + sub + year | age + distance + age × year | 4642.97 | 54.67 |
| age × sub + age × year + juvenile:(band + hatch) | age + sub + year | age + distance + age × year | 4643.46 | 55.15 |

^a^ Difference between a given model’s QAIC_c_ and the lowest within that step.

^b^ Age – juvenile (0–1 year post-hatch) and adult (1+ years post-hatch) rates differ (a ‘:’ indicates that the covariate(s) affect only that age-class); sub – survival and transition rates differ by subpopulation; year – survival and transition rates differ by year; band – age at banding (in days, affected juvenile birds only and appeared in all models) effect on survival; hatch – hatch date (in days, affected juvenile birds only and appeared in all models) effect on survival; high flow (survival) – 2 variables for the effects of (*i*) the high flow events on survival (from 2010–2011) at the high flow Missouri River site (M1F) and (*ii*) the immediate post-high flow environment on M1F survival (from 2011–2012); high flow (transition) – 3 variables for the effects of (*i*) high flow on emigration from M1F to the other areas (from 2010–2011), (*ii*) high flow on immigration into M1F from the other areas (from 2010–2011), and (*iii*) the post-high flow environment on the immigration of individuals into M1F from the other areas (from 2011–2012); success – the effect site-specific reproductive output (chicks fledged/pair) on survival and transition (affected only adult birds for survival); and distance – the effect of the distance among subpopulations on transition rates.
