# Supplementary material for: Metapopulation viability of an endangered shorebird depends on dispersal and human-created habitats: piping plovers (Charadrius melodus) and prairie rivers
Source: Mov Ecol. 2016 Mar 15;4:6. doi: 10.1186/s40462-016-0072-y (PMC4791857; doi:10.1186/s40462-016-0072-y)
Supplement: Additional file 2: — Parameters used in Vortex ver.10 to estimate the population viability of a Great Plains piping plover ( Charadrius melodus ) metapopulation. The metapopulation was composed of three populations located (i) along the lower Platte River (HC, human created habitat) and in the Missouri River on (ii) the Gavins Point Reach (M1F, Missouri River site, high flow) and (iii) Lewis and Clark Lake (M2, Missouri River site, no high flow). File contains all parameters used in Vortex for population viability analysis modeling. (DOCX 98 kb) [file 40462_2016_72_MOESM2_ESM.docx]

Additional File 2. Parameters used in Vortex ver.10 to estimate the population viability of a Great Plains piping plover (*Charadrius melodus*) metapopulation. The metapopulation was composed of three populations located (*i*) along the lower Platte River (HC, human created habitat) and in the Missouri River on (*ii*) the Gavins Point Reach (M1F, Missouri River site, high flow) and (*iii*) Lewis and Clark Lake (M2, Missouri River site, no high flow).

| Parameter | Baseline Value | Description and Notes |
| --- | --- | --- |
| Years | 100 | Population viability was simulated over 100 years. |
| Iterations | 1,000 | Because this is a stochastic model, we ran each simulation 1,000 times to determine average population trends and extinction risk. With 1,000 replications, the risk curves have a 95% confidence interval of about ^+^/- 0.03 (Akçakaya and Root 2005). |
| Time Step | 365 days | Each time step was equivalent to one year. |
| Inbreeding Depression | Not considered | Because a separate study of several piping plover populations throughout North America found that those populations did not exhibit indicators of inbreeding depression (Haig and Oring 1988a), we did not incorporate this parameter in the model. |
| Concordance in Reproduction and Survival? | Yes | We assumed that there was correlation in year-to-year trends in reproduction and survival. |
| Correlation between Populations | 0 | There is no evidence that environmental variability is correlated among populations, likely given the large distances between them (30-200 km; Catlin unpublished data). |
| ***Dispersal (D)*** | | |
| Dispersal Age | 1-14 years | There is no evidence that only certain age groups disperse (Catlin unpublished data). |
| % Survival of Dispersers | 100% | We assumed that all dispersers make it safely to the destination population, where all individuals are subject to age-based mortality. |
| Dispersing Sexes | Males and Females | There is no evidence for sex-dependent dispersal (D. Catlin, unpublished data). |
| Dispersal Modifier | D * [((A<= 1) * 2.3) + ((A > 1) * 1)] | According to the results presented in this paper, hatch years (age 0-1) move on average 2.3 times more often between populations in the study metapopulation than adults. The equation used in the model states that, if an individual is one year old or younger, the probability of dispersal is 2.3 times the dispersal rate calculated according to the equation below, whereas, if the individual is older than one year, the calculated dispersal rate applies. |
| Dispersal Rates | **HC – M1F**: D = [(CAT(1) > 1) * 1.7] + [(CAT(1) = 1) * 4.1] + [(CAT(1) < 1) * 0.2]  **M1F - HC**: D = [(CAT(1) > 1) * 1.6] + [(CAT(1) = 1) * 1.8] + [(CAT(1) < 1) * 5.9]  **M1F - M2**: D = [(CAT(1) > 1) * 8.4] + [(CAT(1) = 1) * 9.8] + [(CAT(1) < 1) * 31.5]  **M2 - M1F**: D = [(CAT(1) > 1) * 7.8] + [(CAT(1) = 1) * 20.4] + [(CAT(1) < 1) *0.9]  **M2 – HC**: D = 0.8  **HC – M2**: D = 0.9 | According to the results presented in this paper, immigration and emigration rates were population-dependent and varied depending on the time since a high flow event has occurred. Therefore, we modeled dispersal from, for example, HC to M1F at (*i*) 1.7% of the population when there had been more than one year since a high flow event, (*ii*) 4.1% when a high flow event occurred in the previous year, and (*iii*) 0.2% in a year that a high flow event occurred. Because floods do not impact habitat availability or dispersal for M2 and HC (results presented in this paper), we did not make dispersal between these populations flow-dependent. We also did not allow for dispersal into saturated populations (where extra individuals would be immediately culled at the carrying capacity). |
| ***Reproductive System and Rates*** | | |
| Reproductive System | Monogamous | Under monogamous mating, there must be one male for each female for breeding to occur. Pairs remain the same within a given year but can change from year to year. Piping plovers are largely monogamous, with rare instances of within-year mate switching (Haig and Oring 1988b). |
| Age of First Offspring (males and females) | 1 year | Both males and females can begin breeding after fledging (Elliot-Smith and Haig 2004). |
| Maximum Age of Reproduction and Lifespan | 14 years | Birds as old as 14 years have been found in the study metapopulation, and there is no evidence of reproductive senescence in this species (D. Catlin, unpublished data). |
| Maximum Number of Progeny | 4 offspring | Pairs produce up to four eggs per brood per year (Elliot-Smith and Haig 2004). |
| Brood Size | 4 offspring | Although some pairs have been observed with two or three eggs in a nest, the vast majority of pairs are observed with four eggs (Elliot-Smith and Haig 2004). Furthermore, it is unclear whether nests observed with less than four eggs actually produced four eggs but lost some due to predation. Therefore, we assumed that all breeding pairs produce four offspring per nest per reproductive event and then allowed the high age-specific mortality observed for hatch years to reduce average yearly productivity accordingly. |
| Distribution of Broods per Breeding Female per Year | | |
| 0 Broods | **HC**: 34.9%  **M1F**: 37.0%  **M2**: 43.5% | Although this species is capable of producing two broods in a single nesting season, the second brood is typically a re-nest after the first nest fails, and most pairs only produce one successful brood in a given year (Elliott-Smith and Haig 2004). The rates, based on the results presented in this paper, were calculated according to Cowardin and Johnson (1979). |
| 1 Brood | **HC**: 65.1%  **M1F**: 63.0%  **M2**: 56.5% |  |
| Sex Ratio at Birth | 50% males | There is no evidence of a sex bias at birth for this species. |
| % Adult Females Breeding | 100% | Current studies of the Great Plains population typically census only the breeding birds, and the initial population sizes used in this model reflect the number of adult breeders. Furthermore, the number of non-breeding females in the population is unknown. Therefore, we assumed that 100% of adult females breed each year, with a fluctuation of 10% around the mean to account for environmental variability. The percentage of breeding females in the M1F population is reduced to 0% during high flow years (see parameterization for Catastrophes below). |
| % Adult Males Breeding | 100% | We assumed that all adult males are capable of breeding in a given year. |
| Density Dependent Reproduction | Not incorporated in model | We assumed that density dependence did not impact reproduction in this metapopulation. Although there is evidence of density dependent effects on reproduction for this species, the mechanisms and total impacts are not clear at this time. |
| ***Mortality*** | | |
| Hatch Year (0-1 year)  Adult (> 1 year) | **HC**: 65.7%  **M1F**: M = [(CAT(1) < 1) *100] + [((CAT(1) >= 1) AND (CAT(1) < 4)) * 56.5] + [((CAT(1) >= 4) AND (CAT(1) < 20)) * (51.1 + (1.8 * (CAT(1)))] + [(CAT(1) >= 20) * 87.1]  **M2**: 74.1%  **HC**: 30.0%  **M1F**: M = [(CAT(1) < 1) *35.5] + [(CAT(1) >= 1) * 27.3]  **M2**: 26.7% | Vortex models mortality as the percentage of individuals of age X that die before reaching age X+1. Values used here are the average mortality rates for the three study populations from 2008 to 2012 (results presented in this paper). Because there is relatively high year-to-year variability in environmental conditions, we varied each mortality rate by +/- 20% of the mean to account for environmental variation. For the M1F population, mortality for both hatch years and adults was influenced by the time since a high flow event had occurred. During a high flow year, adult and hatch year mortality rates increase to 35.5% and 100%, respectively. For all non-high flow years, adult mortality returns to a baseline level of 27.3%. However, for hatch years, mortality rates decline to 56.5% for the 3 years immediately following a high flow event, likely due to increased foraging habitat following the disturbance. This 3-year window of low mortality following a high flow event was very recently confirmed by an ongoing study of that region (K. Hunt unpublished data). After the 3-year window, hatch year mortality increases linearly from 58.3% in year 4 to a high of 87.1% at 20 years post-flood, where mortality rates remain until the next high flow event. According to the results presented in this paper, mortality rates do not change as a function of flows for HC and M2. In the model, mortality is applied before dispersal, and individuals that dispersed to other populations have a higher probability of surviving in the year of a high flow event than those that remain near the M1F population. |
| ***Catastrophes*** | | |
| Type  Global or Local?  Frequency  Severity | High flow event  Global  5%  Impact on survival (M1F only) given in mortality rate. A flood forces reproduction to 0 for M1F during the high flow year. | We modeled the impact of high flows on the study metapopulation. A single high flow event was modeled as global in that it impacted all populations in the same year; however, in this model, high flows only changed (*i*) the immigration rates from M1F to HC and M2, (*ii*) the emigration rates from HC and M2 into M1F, (*iii*) reproduction and survival in M1F, and (*iv*) carrying capacity in M1F. In general, a high flow event submerges all sand bars available to the M1F population, forcing individuals to disperse from that population in that year. Those individuals then must immigrate at an increased rate into the other populations while dispersers from other populations are unable to immigrate into M1F (thus reducing emigration from those populations). In addition, although individuals in the M1F population can fly away from innundated sand bars, those that remain near the M1F population in alternative habitat are subject to higher mortality rates and do not breed in that high flow year. The majority of the HC population is found off-river (around local sand/gravel mines and housing communities), and M2 is heavily managed above the Gavins Point Dam. Therefore, high flow events do not impact the HC and M2 populations in the same manner as they do the M1F population, and we parameterized the model accordingly. We assumed that high flows of this magnitude occur every 20 years on average (based on historical discharge rates from Gavin’s Point Dam, available at http://www.nwd-mr.usace.army.mil/rcc/projdata/gapt.pdf). |
| ***Initial Population Size (N_0_)*** | **HC**: 126 individuals  **M1F**: 421  **M2**: 106 | Values based on a census of the breeding population in 2008 by the study authors. We assumed that the population was within a stable age distribution. |
| ***Carrying Capacity (K)*** | **HC**: 135 individuals  **M1F**: K =((Y=0) * 804) + ((Y>0) * (((CAT(1)=0) * PS1) + ((CAT(1)=1) * 2154) + (((CAT(1) >1) AND (CAT(1) < 100)) * (PS1 - (PS2 * PS1))) + ((CAT(1) >= 100) * (PS1 - (PS2 * PS1)))))  **M2**: 98 individuals  **PS1 = previous year’s K  ** PS2 = rate of habitat loss (10-60%) for that year as randomly chosen by *Vortex*  PS2 = (0.5 *(RAND))+0.1) | For HC, carrying capacity (K) estimates were derived from expert opinion and observations of the population from 2008-2013 (M. Bomberger-Brown unpublished data). Using observations from 2007 to 2011 for M2, we multiplied the area of suitable habitat observed each year by a previously observed subpopulation-wide density (0.7 pairs per ha; J. Fraser, unpublished data) and multiplied that value by two to approximate the number of birds that the area could support each year. We then found the average and standard deviation of these yearly values to determine the average K and by how much that K fluctuates due to environmental variation. Because K is influenced by high flows for the M1F population, we made carrying capacity and its fluctuations due to environmental variation a function of the time since a high flow event had occurred. We started the model such that the K for this population was 804 individuals. For the remainder of the simulation, K (*i*) remains at the previous year’s level in the year that a high flow event occurs, (*ii*) increases to 2154 individuals in the year after a high flow event occurs, based on the observed increase in habitat following the 2011 high flow event (U.S. Army Corps of Engineers [USACE], unpublished data) and (*iii*) declines from 2154 by 10-60% each year due to erosion and vegetation encroachment until the next high flow event occurs, based on empirical observations from a high water event in the late 1990s (USFWS 2009, USACE unpublished data). These values were calculated according to the same methodology used to calculate K for the M2 population, using observations of the area of suitable sand bar habitat from 1997 to 2012 (Catlin et al. 2015) and assuming a density of 0.7 pairs per ha. In the high flow year, K would technically decline to zero for that year. However, in *Vortex*, all individuals who did not disperse would be culled, which is not realistic (individuals move temporarily but do not die). Therefore, we maintained K in the high flow years at the level observed in non-high flow years, but we reduced reproduction for that year to 0. |
